# TatS: a novel in vitro tattooed human skin model for improved pigment toxicology research

**DOI:** 10.1007/s00204-020-02825-z

**Published:** 2020-07-13

**Authors:** Henrik Hering, Christian Zoschke, Markus Kühn, Ashish K. Gadicherla, Günther Weindl, Andreas Luch, Ines Schreiver

**Affiliations:** 1 Department of Chemical and Product Safety, German Federal Institute for Risk Assessment (BfR), Berlin, Germany; 2 Institute of Pharmacy (Pharmacology and Toxicology), Freie Universität Berlin, Berlin, Germany; 3 Department of Biological Safety, German Federal Institute for Risk Assessment (BfR), Berlin, Germany; 4 Section of Pharmacology and Toxicology, Pharmaceutical Institute, University of Bonn, Bonn, Germany

**Keywords:** Rutile, Anatase, Carbon black, Pigment Orange 13, Tissue engineering, Tattooing

## Abstract

Reports of tattoo-associated risks boosted the interest in tattoo pigment toxicity over the last decades. Nonetheless, the influence of tattoo pigments on skin homeostasis remains largely unknown. In vitro systems are not available to investigate the interactions between pigments and skin. Here, we established TatS, a reconstructed human full-thickness skin model with tattoo pigments incorporated into the dermis. We mixed the most frequently used tattoo pigments carbon black (0.02 mg/ml) and titanium dioxide (TiO_2_, 0.4 mg/ml) as well as the organic diazo compound Pigment Orange 13 (0.2 mg/ml) into the dermis. Tissue viability, morphology as well as cytokine release were used to characterize TatS. Effects of tattoo pigments were compared to monolayer cultures of human fibroblasts. The tissue architecture of TatS was comparable to native human skin. The epidermal layer was fully differentiated and the keratinocytes expressed occludin, filaggrin and e-cadherin. Staining of collagen IV confirmed the formation of the basement membrane. Tenascin C was expressed in the dermal layer of fibroblasts. Although transmission electron microscopy revealed the uptake of the tattoo pigments into fibroblasts, neither viability nor cytokine secretion was altered in TatS. In contrast, TiO_2_ significantly decreased cell viability and increased interleukin-8 release in fibroblast monolayers. In conclusion, TatS emulates healed tattooed human skin and underlines the advantages of 3D systems over traditional 2D cell culture in tattoo pigment research. TatS is the first skin model that enables to test the effects of pigments in the dermis upon tattooing.

**Electronic supplementary material:**

The online version of this article (10.1007/s00204-020-02825-z) contains supplementary material, which is available to authorized users.

## Introduction

Tattooing is an ancient body modification in which sharp tools are used to insert non-soluble pigments into the dermal layer of the skin. After healing, the tattoo pigments reside in the dermis where they can be taken up by fibroblasts and macrophages (Anderson et al. 1996; Zelickson et al. 1994). Pigments are also transported to the lymph nodes and likely to other organs (Schreiver et al. 2017).

Within the last decades, the prevalence of tattooing increased in Western societies. Up to 44% of people in the age group around 30 have at least one tattoo (Borkenhagen et al. 2019; Kluger 2015). With an increasing frequency of tattooing, the overall number of observed tattoo-associated side effects also increased. Besides phototoxicity, which represents 60% of side effects (Hutton Carlsen and Serup 2014), the contamination and release of potential carcinogens and other toxic substances are possible risks related to tattooing (Hauri and Hohl 2015; Hering et al. 2018; Schreiver et al. 2016). In addition, infections, allergies and foreign body granulomas are also common (Serup et al. 2016).

An appropriate test system to evaluate the toxicological effects of tattoo pigments is still missing. Conventional 2D cell cultures suffer from several shortcomings. Due to agglomeration and sedimentation of tattoo pigments, regular cell growth is negatively affected and dosage comparability between human tissues and 2D cultures remains uncertain (Allouni et al. 2009; Cho et al. 2011; Wu et al. 2017). The lack of extracellular matrix and other histological features present in natural human skin compromises the applicability of 2D cell cultures for tattoo pigment research. Animal testing on the other hand is expensive, controversial due to ethical concerns and the intradermal dosage during tattooing is hard to control. 3D reconstructed skin models are commercially available and already used to investigate the sensitizing properties of tattoo inks (Bil et al. 2018). Due to its water insolubility, however, tattoo inks can be only suspended in a vector liquid made of manifold substances including solvents, dispersants, preservatives and fragrances. Transferring the ink into the medium will, therefore, only mirror the toxicity of soluble ingredients. On the other hand, tattooing of commercially available 3D reconstructed skin models might significantly damage the tissue and the healing time of the models most likely exceeds the possible culture periods. Last but not least, dosimetry of tattoo pigments in 3D culture is as difficult as in animal models.

An ideal in vitro test system for studying possible adverse effects of tattoo pigments in human skin has to fulfill the following basic features: (i) possibility to culture living skin cells along with tattoo pigments; (ii) enabling an accurate dosage of tattoo pigments; (iii) culturing in a 3D tissue-like environment; and (iv) presence of a skin-like extracellular matrix.

Therefore, we chose to add a defined amount of tattoo pigments during production of the dermal layer of TatS. For this study, we selected four different pigments to investigate their possible effects in human skin. Carbon black was chosen since it is the most frequently used tattoo pigment. White titanium dioxide (TiO_2_) is frequently used to blend different color shades. Both, its anatase and rutile crystal structures were investigated due to their use in tattoo inks and the expected differences in their toxicological properties. Also the organic diazo compound Pigment Orange (P.O.) 13 was included in this study since its cleavage products were shown to be (geno-)toxic to human skin cells (Hering et al. 2018).

TatS was characterized by investigating its histological features compared to tattooed human skin and other full thickness skin models. Due to the potential role of interleukin (IL)-6 and IL-8 in sarcoidosis (Denisova et al. 2013) and skin inflammation, we analyzed the secretion of these cytokines in TatS and normal human dermal fibroblasts (NHDF). To include keratinocyte secreted cytokines involved in skin inflammation, we also analyzed IL-1α, granulocyte-colony stimulating factor (G-CSF), granulocyte–macrophage colony-stimulating factor (GM-CSF), and transforming growth factor alpha (TGF-α). IL-18 was included due to its current application as putative biomarker of tattoo-related skin sensitization (Bil et al. 2018). Our goal was to establish viable TatS that resemble fully healed tattooed human skin. We also compared TatS with 2D culture systems to evaluate whether advantages exist that would justify the application of the more complex 3D models in tattoo pigment research.

## Materials and methods

### Materials

Sodium hydroxide (NaOH), trypsin inhibitor (from *Glycine max*), 3,3′,5-triiodo-L-thyronine sodium salt, hydrocortisone, cholera toxin, epidermal growth factor (EGF, recombinant human protein), insulin, transferrin (human), Hoechst 33258, sodium dodecyl sulfate (SDS), glycine, adenine hydrochloride hydrate, uranyl acetate, propylene oxide and l-ascorbic acid were purchased from Sigma-Aldrich (St. Louis, MO, USA). PrestoBlue cell viability reagent, SuperFrost Plus microscope slides, Dulbecco's Modified Eagle Medium: Nutrient Mixture F-12 (DMEM/F-12) (no phenol red), E-cadherin monoclonal antibody (4A2C7, cat. 33-4000), collagen IV monoclonal antibody (1042, cat. 14-9871-82), occluding polyclonal antibody (6HCLC, cat. 710192), goat anti-mouse IgG (H + L) cross-adsorbed secondary antibody (alexa fluor 546 and alexa fluor 488), goat anti-rabbit IgG (H + L) cross-adsorbed secondary antibody (alexa fluor 546 and alexa fluor 488), ProLong diamond antifade mountant, tris(hydroxymethyl)aminomethane (Tris), SuperSignal West Dura Extended Substrate and MEM non-essential amino acids solution (100x) were purchased from Thermo Fisher Scientific Inc. (Waltham, MA, USA). Fetal bovine serum superior (FBS), 10x DMEM solution, 4-(2-hydroxyethyl)-1-piperazine ethanesulfonic acid (HEPES) buffer (1 M) and Collagen G (0.4% solution in 15 mmol/l HCl, type I, 4 mg/ml) were purchased from Biochrom (Berlin, Germany). Triton X-100, urea and dimethyl sulfoxide were purchased from Carl Roth (Karlsruhe, Germany). ThinCert cell culture inserts for 12-well plates (translucent membrane (polyethylene terephthalate), pore diameter: 8 µm) and 12-well ThinCert cell culture plates (deep-well) were purchased from Greiner Bio-One (Kremsmünster, Austria). GA-1000 (gentamicin sulfate amphotericin-B, 1000 x) was purchased from Lonza (Basel, Switzerland). Dulbecco's phosphate-buffered saline (DPBS), trypsin/ethylenediaminetetraacetic acid (EDTA) (0.05%/0.02%) in PBS, l-glutamine (200 mM) and penicillin/streptomycin (100 U/ml) were purchased from PAN-Biotech GmbH (Aidenbach, Germany). Tissue-Tek cryomolds (15 mm × 15 mm × 5 mm) and Tissue-Tek O.C.T. compounds were purchased from Sakura Finetek (Torrance, CA, USA). Dispase (1 U/ml) in DMEM/F-12 was purchased from Stemcell Technologies (Cologne, Germany). Keratinocyte Growth Medium 2 (kit, growth medium supplemented with final concentrations of: 0.004 ml bovine pituitary extract per ml final medium, 0.125 ng/ml EGF (recombinant human), 5 μg/ml insulin (recombinant human), 0.33 μg/ml hydrocortisone, 0.39 μg/ml epinephrine, 10 μg/ml transferrin (recombinant human), 0.06 mM CaCl_2_) was purchased from PromoCell (Heidelberg, Germany). Tenascin C monoclonal antibody (EB2, cat. ab88280) was purchased from Abcam (Cambridge, UK). Filaggrin monoclonal antibody (FLG/1562, cat. NBP2-53245-20 μg) was purchased from Novus Biologicals (Littleton, CO, USA). Glutaraldehyde and osmium tetroxide solution were purchased from Electron Microscopy Sciences (Hatfield, PA, USA). Gelatin capsules were purchased from Parke Davis (Detroit, MI, USA). EPON resin mixture was purchased from Agar Scientific (Stansted, UK). Lead citrate was purchased from Ferak Berlin GmbH (Berlin, Germany).

The pigments used in this study were Irgalite Orange D2895 (P.O.13, C.I. 21110, 4,4′-[(3,3′-dichloro[1,1′-biphenyl]-4,4′-diyl)*bis*(azo)]*bis*[2,4-dihydro-5-methyl-2-phenyl-3*H*-pyrazol-3-one)]) provided from BASF (Ludwigshafen, Germany), TiO_2_ nanopowder rutile (high purity, 99.9%, 30 nm, stock #: US3520) and anatase (high purity, 99.98%, 30 nm, stock #: US3498) purchased both from US Research Nanomaterials Inc. (Houston, TX, USA). ENSACO® 250P (carbon black, C.I. 77266, polycyclic aromatic hydrocarbons content below 1 ppm) was provided from Imerys Graphite & Carbon (Bironico, Switzerland).

### Isolation of primary human skin cells from neonatal foreskin

Normal human keratinocytes (NHK) and NHDF were isolated from neonatal foreskin derived from therapeutically indicated circumcisions after written informed consent of the legal guardian (ethical approval EA2/104/18, Charité Berlin and ethical approval Eth-17/19, Berlin Chamber of Physicians).

Human foreskin tissue was transported in DPBS at 4 °C to the BfR. The foreskin tissue was then washed with DPBS. Vascular, mucosal and adipose tissue was removed using scalpels and/or scissors. Afterwards, the foreskin tissue was stretched on autoclaved metal gaze, the dermal side facing toward the metal gaze, and placed in a 6 cm dish containing dispase solution with the epidermis faced downwards and incubated overnight at 4 °C.

The next day, the epidermis was removed using forceps and incubated in trypsin/EDTA solution for 20 min at 37 °C to isolate NHK. Trypsin reaction was stopped using trypsin inhibitor (1 mg/ml) in DPBS. NHK were then cultured in keratinocyte growth medium 2.

To obtain NHDF, residual dermal tissue was cut into 3 mm pieces and put face down into 6 cm dishes containing minimal medium (DMEM/F12 without phenol red supplemented with 10% FBS, 1% penicillin/streptomycin and 0.1% GA-1000). NHDF were allowed to grow out of the tissue for 1 week. Both NHK and NHDF were passaged once followed by cryopreservation in DMEM/F12 with 10% FBS and 10% DMSO (passage = 2) until usage.

Isolated cells and dermal pieces in minimal medium were cultured at 37 °C, 5% CO_2_ atmosphere and 90% relative humidity.

### Production of TatS

To produce tattooed reconstructed human skin equivalents (TatS) with and without tattoo pigments, we modified an existing protocol described elsewhere (Zoschke et al. 2016). We included tattoo pigments into the buffer used to create the dermal part of TatS. Several concentrations of pigments in the dermis were tested, starting with 0.4 mg/ml per pigment and decreased, if dermis formation was prevented, as seen by inhibited dermis shrinking. In detail, tattoo pigments were suspended in tenfold concentrated dermis buffer (50% 10 × DMEM, 25% NaOH in H_2_O, 25% HEPES). The pigment suspensions were then vortexed rigorously and ultra-sonicated for 25 min in a Sonopuls HD 2200 from Bandelin electronic (Berlin, Germany). Afterwards, 3 × 10^5^ NHDF were embedded in a collagen matrix (80% collagen G solution, 10% dermis buffer with suspended tattoo pigments and 10% MM). 900 µl of this mixture was poured into a THINCERT trans-well placed in a THINCERT 12-deep-well-plate (both from Greiner Bio-One International GmbH, Kremsmünster, Austria) on top of the cell-free collagen and incubated for 45 min in the cell CO_2_ incubator. Subsequently, additional 250 µl of pigment-free collagen matrix with NHDF were given on top of the pigmented layer, forming a second pigment-free layer. After another incubation for 2 h, 500 µl dermal growth medium (minimal medium supplemented with 40 µM adenine hydrochloride monohydrate, 30 µg/l amphotericin B, 0.1 nM cholera toxin, 10 µg/l EGF, 3.5 mg/l hydrocortisone, 4.4 mg/l insulin, 0.5% non-essential amino acids, 4.4 mg/l transferrin, and 2 nM triiodothyronine) were given on top of the dermal layers and 4 ml into the deep well. Medium was changed twice before seeding the keratinocytes.

One week after construction of the dermal layers, medium in the trans-well was aspirated. 1 × 10^6^ NHK (passage = 3) was poured onto each dermal layer. On the consecutive day, an airlift was performed: the remaining medium in the trans-wells was aspirated and medium in the wells was changed. Dermal growth medium was replaced by epidermal growth medium (dermal growth medium supplemented with 0.25 mM ascorbic acid and 1.8 mM calcium chloride). Epidermal growth medium was changed three times a week. TatS were incubated for two additional weeks until full differentiation of the epidermal layer.

### Histological characterization of TatS

To compare general histological features and especially epidermal differentiation, we used haematoxylin–eosin (H&E) stain and immunofluorescence staining. At the end of culture, TatS were snap-frozen in Tissue-Tek cryomolds containing Tissue-Tek O.C.T. compound using liquid nitrogen. Consecutively, the embedded tissue sections were packed with laboratory film and aluminum foil and stored at − 80 °C until sectioning.

Cryopreserved TatS were sectioned using the cryostat Microm HM 550 (Thermo Fisher Scientific, Waltham, MA, USA) with a thickness of 5–7 µm and mounted on SuperFrost slides (Thermo Fisher Scientific). In the cryo block, each TatS was oriented vertically toward the microtome knife to prevent translocation of the pigments to other skin layers due to sectioning and in a way that a depth profile from *stratum corneum* to dermis is given. Mounted sections were stored at − 80 °C till H&E or immunofluorescence staining.

For immunofluorescence staining, the slides were fixed in 100% ice-cold methanol. Methanol was allowed to vaporize for 5 min at room temperature (RT) and the slides were consecutively washed and rehydrated using DPBS. TatS sections were then permeabilized using 0.5% Triton X-100 in DPBS for 15 min. Afterwards, the slides were washed again two times with DPBS and then blocked with the blocking solution containing 5% FBS and 0.1% Triton X-100 in DPBS (PBST) for 30 min at RT. Afterwards, the respective primary antibody solution was poured onto the slides and incubated in a dark chamber at 4 °C over night. TatS sections were stained with the following primary antibodies: E-cadherin monoclonal antibody (5 µg/ml), occludin recombinant polyclonal antibody (5 µg/ml) and filaggrin monoclonal antibody (4 µg/ml) to visualize epidermal development; collagen IV monoclonal antibody (5 µg/ml) and tenascin C (0.1 µg/ml) to visualize basal membrane development. After staining with primary antibodies, slides were washed two times with PBST followed by 45 min incubation with the correspondent secondary antibody (1:400 in PBST). For TatS sections with incorporated P.O.13 pigments, secondary antibodies coupled with alexa flour 488 instead of 546 were used to avoid interferences with the auto-fluorescence of P.O.13. Afterwards, slides were washed two times with PBST and once with DPBS for 5 min each. Sections were then incubated for 15 min with Hoechst 33258 (1 µg/ml in DPBS) and then washed again two times with DPBS. Residual DPBS was carefully removed. Slides were then covered with ProLong diamond antifade mountant and analyzed using an LSM700 confocal microscope from Carl Zeiss (Oberkochen, Germany). Negative controls were processed omitting the primary antibody.

### Transmission electron microscopy

To analyze the shape and primary particle size of tattoo pigments and to investigate the possible uptake of the pigments by fibroblasts, we performed transmission electron microscopy (TEM).

At the end of culture, TatS were sectioned into 2 × 4 mm cuboids containing only the dermal layer with incorporated pigments. The cuboids were then placed in a 1.5 ml reaction tube and fixed with glutaraldehyde for 24 h. Following fixation, the cuboids were transferred into gelatin capsules and washed three times with HEPES buffer (0.2 M HEPES, pH 7.2). The cuboids were then incubated for 2 h in osmium tetroxide solution, followed by a 30 min wash in HEPES buffer. The blocks were then contrasted with uranyl acetate (0.6% w/v in H_2_O). Following dehydration using increasing amounts of ethanol, the blocks were transferred into a mixture of 96% ethanol, 4% propylene oxide for 30 min followed by 1 h in propylene oxide–Epon resin mixture. The solution was then removed, fresh Epon resin added to the gelatin capsule, and polymerized for 24 h each at 30 ºC, 40 ºC and 60 ºC. The blocks were cut into 70–80 nm ultrathin sections and transferred onto 200 mesh copper grids (Plano GmbH, Wetzlar, Germany). The sections on copper grids were further stained with lead citrate to achieve dual contrast.

Grids were then observed in a Jeol 1400plus TEM (Jeol, Japan) operated at 120 kV. Imaging was performed using a Veleta G2 camera and iTEM software (both from Olympus Europa SE & Co. KG, Hamburg, Germany).

### Treatment of NHDF with tattoo pigments

NHDF were seeded in a 24-well plate at a concentration of 5 × 10^4^ cells/well and treated with tattoo pigments at concentrations comparable to those used in TatS in terms of the amount of pigments per cell (NHDF). Pigments per cell ratio in TatS and the 2D experiment were 1.33 ng per cell for TiO_2_ anatase and TiO_2_ rutile, 0.67 ng per cell for P.O.13, and 0.067 ng per cell for carbon black, respectively. Final concentrations used to treat NHDF with pigments were 66 µg/ml for TiO_2_ anatase and TiO_2_ rutile, 33 µg/ml for P.O.13 and 3.3 µg/ml for carbon black.

Tattoo pigment suspensions in minimal medium were prepared as twofold concentrated with ultra-sonication as described above. In case of P.O.13, minimal medium with 0.01% Triton X-100 was used for the stock suspension (with a final Triton X-100 concentration below 0.001%). NHDF suspensions at a concentration of 1 × 10^5^ cells/ml and pigment suspension were mixed 1:1 and then seeded in a 24-well plate.

### PrestoBlue viability assay

Viability of TatS and NHDF was assessed using the PrestoBlue assay. PrestoBlue assay is a resazurin-based non-disruptive cell viability assay that bears the advantage to use TatS after assessment of viability for other purposes, like immunofluorescence (Young and Reed 2016). Three TatS with pooled cells from three donors were used. For NHDF, each replicate was done with cells from a different donor.

In case of TatS, 500 µl PrestoBlue solution (10% in MM) was put on TatS and incubated at 37 °C for 1.5 h. In case of NHDF, 300 µl of a 10% PrestoBlue solution in fibroblast growth medium was put on NHDF and incubated at 37 °C for 2 h. Afterwards, fluorescence in supernatants was measured using a Synergy HT-reader (BioTek Instruments GmbH, Bad Friedrichshall, Germany) at an excitation wavelength of 530 nm and an emission wavelength of 590 nm.

### Cytokine release

To analyze cytokine release from TatS and NHDF, we quantified G-CSF, GM-CSF, IL-1α, IL-6, IL-8, IL-18 and TGF-α using a custom LEGENDplex Multi-Analyte Flow Assay Kit (Biolegend, San Diego, CA, USA). TatS made of cells from different donors were used for each replicate.

Briefly, NHDF were treated with tattoo pigments as described above and cultured overnight for 16 h. Medium was changed and samples for cytokine quantifications were taken 8 h after. To reach comparable conditions for cytokine detection between TatS and NHDF, 666 µl medium was used, resulting in comparable amounts of medium per NHDF.

For TatS, samples were taken at the end of the culture period at day 21. At day 19, medium was changed from keratinocyte growth medium to minimal medium. Medium was then changed once more before cytokine analysis. At day 20, medium was changed again, and samples were taken 8 h afterwards.

150 µl of medium was transferred into a 1.5 ml reaction tube and centrifuged with 16,100×*g* for 10 min at 4 °C in a 5415 R micro-centrifuge (Eppendorf, Hamburg, Germany) with the rotor FA-45-24-11 to get rid of tattoo pigments and cell debris. The supernatant was then stored at − 80 °C until the bead-based cytokine assay was performed according to the manufacturer’s instructions. The flow cytometer BD FACSARIA III (Becton Dickinson, Franklin Lakes, NJ, USA) was used to read the beads. Blanks and standard curves were included in each experiment. Three independent experiments were carried out for TatS and 2D cells, respectively.

### Microscopy image processing

Microscopic images were processed using ZEN Black 2012 software (Carl Zeiss, Oberkochen, Germany). Pairwise stitching of H&E pictures was performed in Fiji (Schindelin et al. 2012) as described elsewhere (Preibisch et al. 2009).

### Statistics

Data processing was performed with Microsoft Excel (Microsoft, Redmond, WA, USA). Data analysis and illustration were performed using GraphPad Prism 8 (GraphPad Software, San Diego, CA, USA). Presto blue and cytokine data were normalized by dividing each value by the value of the respective pigment-free control of the same donor. Data were assumed to be normally distributed and statistically analyzed by one-way ANOVA with Dunnett's test. A value of *p* ≤ 0.05 was accepted as statistically significant. No experimental data were excluded from statistical analysis.

## Results

### Histological characterization of TatS

**Fig. 1:**
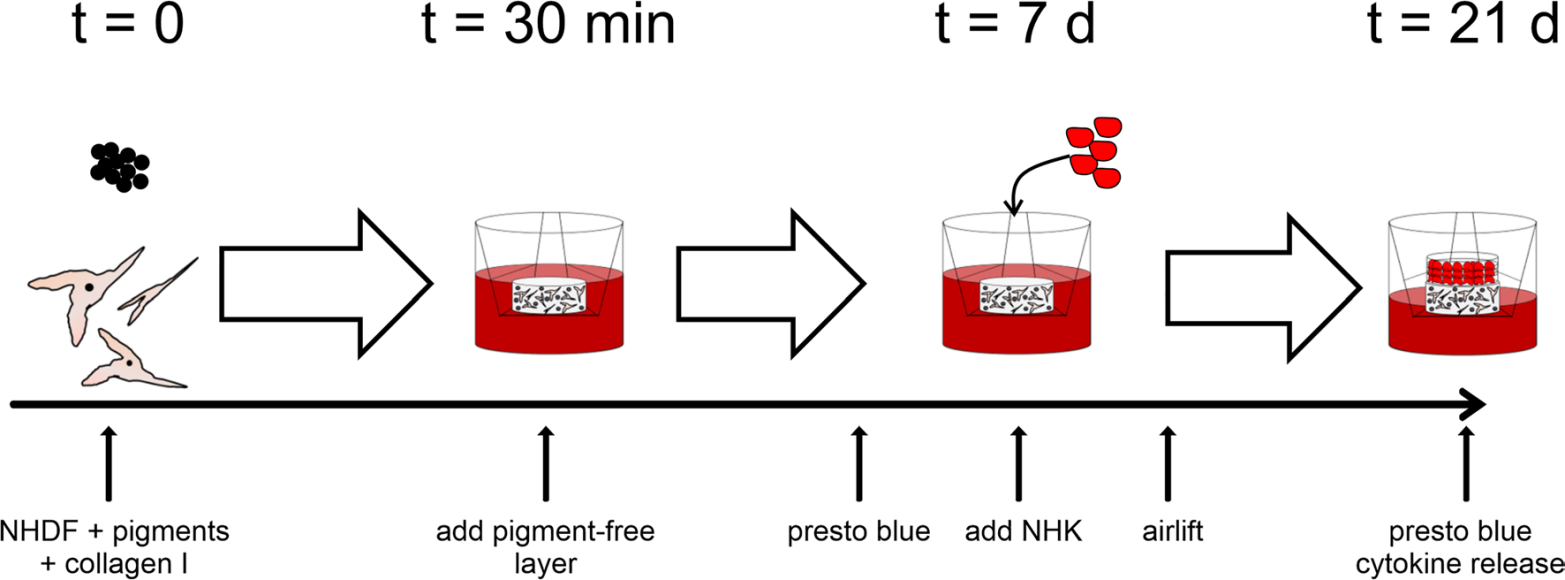
Construction of tattooed human skin models (TatS) and experimental outline. Dermal layers of TatS were produced using pigments, normal human dermal fibroblasts (NHDF), and collagen I. A second layer of collagen I and fibroblasts was added after 30 min. After 7 and 21 days of culture, viability was measured using the presto blue assay. Afterwards, normal human keratinocytes (NHK) were seeded on top of TatS. The day after, an airlift was performed. After another 2 weeks of culture, medium was changed to minimal medium. Two days later, cytokine secretion and viability of TatS were investigated. At day 21, TatS were frozen and sectioned for histological analyses

To create an appropriate 3D skin model that resembles healed tattooed human skin, we incorporated tattoo pigments into the dermal layer during their production (Fig. 1). The initial concentration of pigment in the dermis solution was 0.4 mg/ml unless an inhibition of dermis formation was seen macroscopically by the absence of model contraction during the first week of culture. Thus, final concentrations of tattoo pigments in the dermis were 0.4 mg/ml for TiO_2_ anatase and TiO_2_ rutile, 0.2 mg/ml for P.O.13, and 0.02 mg/ml for carbon black, respectively. All final concentrations led to a macroscopically visible coloration of TatS and were, therefore, supposed to be of acceptable quality (Supplementary Fig. S1).

**Fig. 2:**
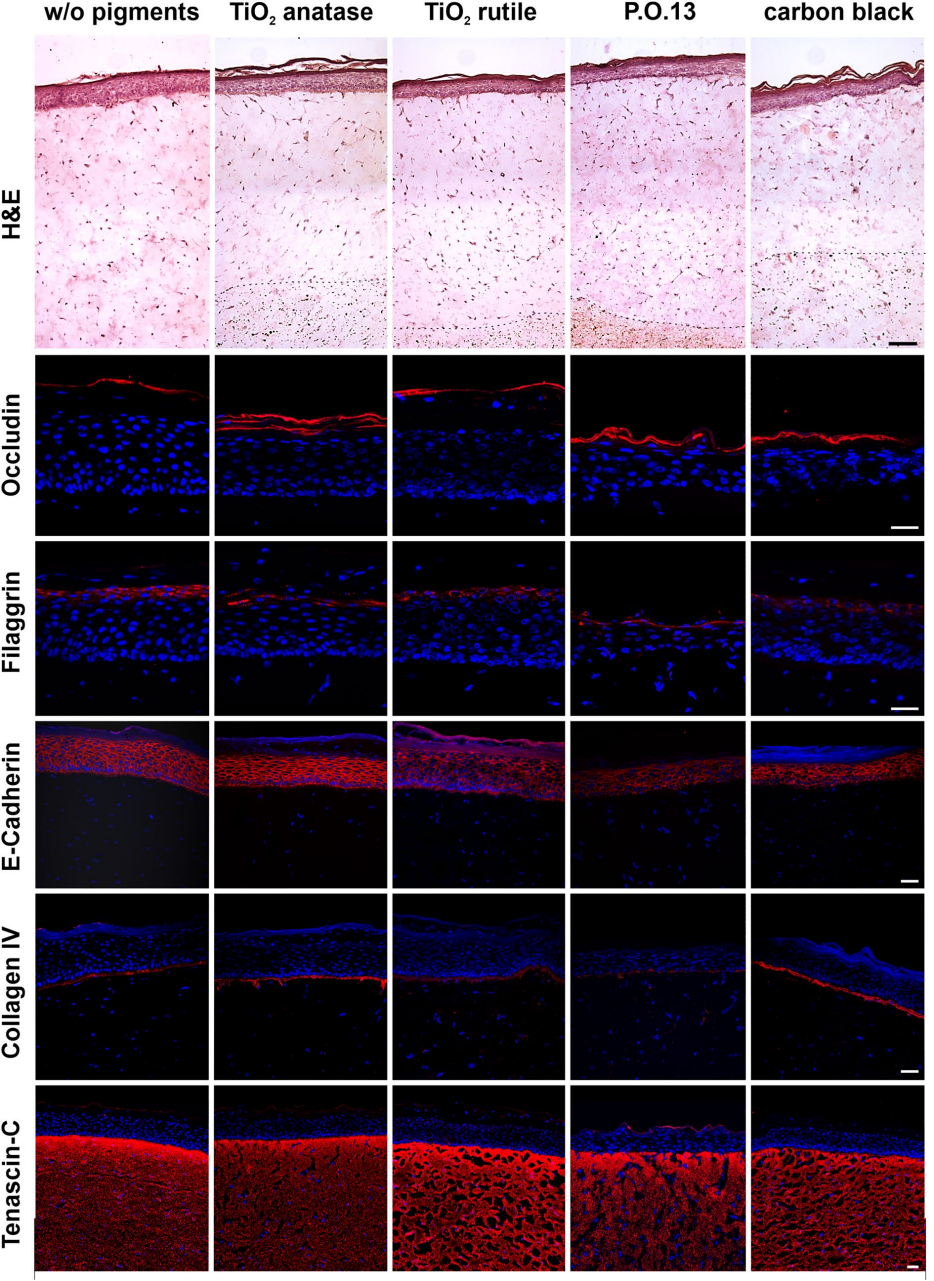
Histological characterization of tattooed human skin models (TatS). The columns show (from top to bottom): haematoxylin and eosin (H&E) stain and immunofluorescence staining of occludin-1, filaggrin, E-cadherin, collagen IV and tenascin-C (all in red). Immunofluorescence pictures were counterstained with Hoechst 33258 (blue), indicating the cell nuclei. Dotted lines in H&E pictures indicate the pigment border. Black bar for H&E stain equals 100 µm, white bars equal 50 µm. w/o, without; titanium dioxide, TiO_2_; Pigment Orange 13, P.O.13

We ensured the comparability to other skin model system by examining the tissue structure of pigmented and pigment-free TatS. Pigment-free dermal layers have a maximum depth around 800 µm, decreasing toward the edges of TatS and are comparable between different pigments (Fig. 2). Smaller volumes of the pigment-free layer led to insufficient covering of the first pigmented dermal layer. H&E staining revealed comparable epidermal thickness and *stratum corneum* development with no ectopic pigment disposition in the epidermis. Previous protocols without a pigment-free dermal layer led to the ectopic disposition of pigments (Supplementary Fig. S2). Basal keratinocytes were well ordered in a single columnar layer (Fig. 2). Keratinocytes above the basal layers were differentiating as judged by its flattening toward the *stratum corneum*. Fibroblast distribution was comparable between pigmented and pigment-free dermis. Occludin-1, filaggrin and E-cadherin staining were identical independent of the pigments used, suggesting similar epidermal differentiation and growth. In all TatS, collagen IV staining revealed the formation of a dermo-epidermal junction, that is, the basement membrane.

While tenascin C staining was similar between all pigments, general dermis integrity upon cryopreservation was slightly reduced in sections of pigmented TatS.

**Fig. 3:**
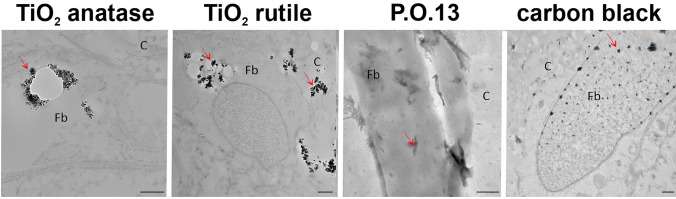
Transmission electron microscopy (TEM) reveals uptake of tattoo pigments by fibroblasts in TatS. TEM was used to investigate tattoo pigment primary particle shapes and sizes in TatS as well as tattoo pigment–cell interactions in TatS. Scale bars equal 500 nm. *w/o* without, *TiO*_*2*_ titanium dioxide, *P.O.13* Pigment Orange 13, *Fb* fibroblast, C collagen matrix

TEM revealed the uptake of pigments into the NHDF of TatS for TiO_2_ anatase and rutile and, to the highest extent, for carbon black (Fig. 3). For P.O.13, no cell cross-section was obtained. However, the pigments seem to reside on the cell surface of NHDF.

Agglomerations of all pigments exceeded the nanorange (> 100 nm) and could be detected by light microscopy (Fig. 2). TiO_2_ anatase and rutile primary particles are nanospheres with a mean diameter in the lower nanometer range around 22 nm and 50 nm, respectively (Fig. 3). P.O.13 pigments appear more rod-shaped than spheric and are in the nano- to micrometer range. Carbon black agglomerates and primary particles remained indistinguishable in the TEM images. However, agglomerates taken up by cells vary in size between 40 nm and 360 nm, with a mean diameter around 85 nm, which is still in the nanorange.

### Impact of pigments on cell viability of NHDF in 2D culture and TatS

To ensure comparable viability of TatS with incorporated tattoo pigments to the corresponding control without tattoo pigments, we performed a PrestoBlue assay on fully grown TatS.

**Fig. 4:**
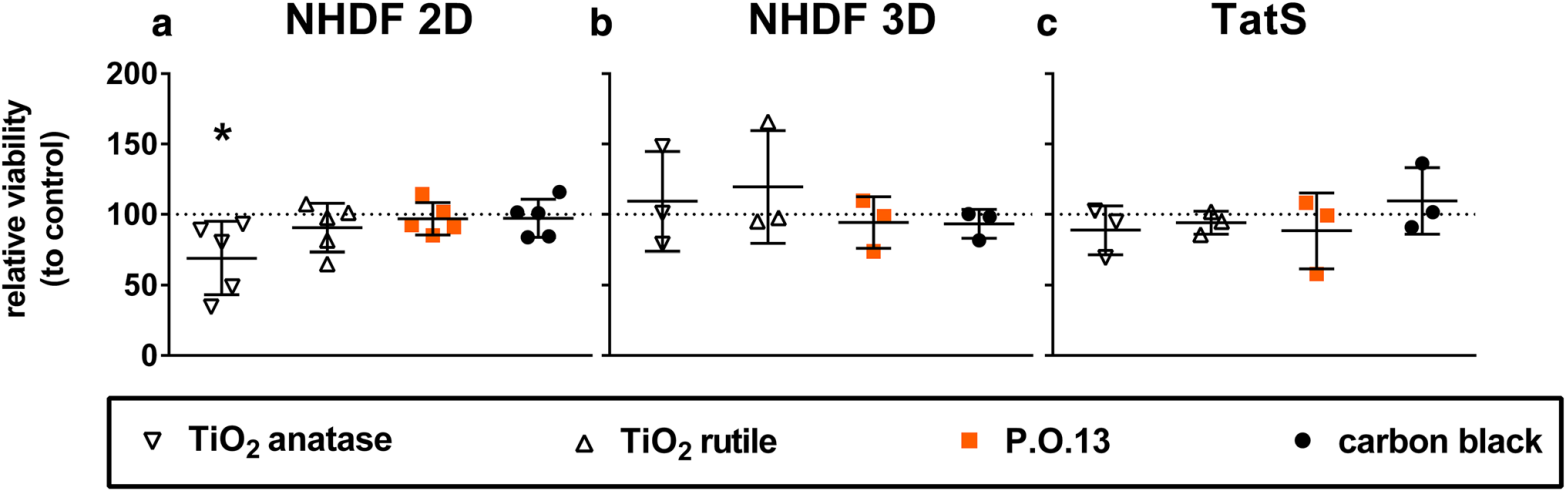
Comparison of the viability of 3D and 2D fibroblast cultures with tattooed human skin models (TatS) upon treatment with tattoo pigments. PrestoBlue assay was performed on **a** normal human dermal fibroblasts (NHDF 2D), **b** dermal layers of tattooed reconstructed human skin (NHDF 3D) or **c** TatS each with pigments in corresponding pigment-to-cell ratios. PrestoBlue assay was performed 24 h after NHDF (**a**) seeding or 7 (**b**) and 21 (**c**) days after construction of dermal layers of the TatS. Data were normalized by dividing each value by the value of the respective pigment-free control of the same donor. Data are displayed as mean ± SD of three dermal layers and three complete TatS from different donors (*n* = 3) and NHDF from five different donors (*n* = 5). Asterisks indicate significance between substance treatments and the respective control as identified by one-way ANOVA with Dunnett’s test (*p < 0.05). TiO_2_ titanium dioxide, *P.O.13* Pigment Orange 13

No significant decrease in tissue viability was detected between TatS with or without tattoo pigments located in the dermis. However, viability of fully-grown TatS varied more for TatS with incorporated carbon black and P.O.13 pigments than for those with TiO_2_ pigments (Fig. 4, Supplementary Table S1).

In fully-grown TatS, dermal layers are covered by epidermal keratinocytes, which outnumber the dermal fibroblasts by far. Therefore, the keratinocytes may mask possible cytotoxic effects of the tattoo pigments on the dermal fibroblasts. To measure the viability of dermal fibroblasts without a possible masking by keratinocytes, we performed PrestoBlue assay on TatS the day before keratinocyte seeding (NHDF 3D).

The viability of NHDF 3D varied insignificantly with all pigments when compared to the corresponding control (Fig. 4b, Supplementary Table S2). However, variations in viability were higher in NHDF 3D with incorporated TiO_2_ pigments than for those with incorporated carbon black and P.O.13 pigments.

To differentiate the effects of tattoo pigments on the viability of skin cells in 2D and 3D, we performed PrestoBlue assay on NHDF treated with tattoo pigments in the same pigment to cell ratio we used in TatS (Fig. 4, Supplementary Table S3). Since 2D cell cultures are not stable for time-periods used for TatS and NHDF 3D experiments, we chose 24 h as standard incubation time for in vitro assays.

From all pigments tested, only TiO_2_ anatase reduced the viability in 2D cell culture of NHDF significantly. The mean viability of TiO_2_ rutile seems to be decreased in 2D. P.O.13 and carbon black induced no significant changes.

### Cytokine release of TatS and NHDF treated with tattoo pigments

Finally, we analyzed the inflammatory response in TatS caused by tattoo pigments by determining the secretion of relevant cytokines. Here, we quantified the secretion of the proinflammatory cytokines IL-1α, IL-6, IL-18, G-CSF and GM-CSF as well as the chemokine IL-8 and the transforming growth factor TGF-α. We additionally quantified cytokine secretion in supernatants of NHDF and compared the results to those of TatS.

**Fig. 5:**
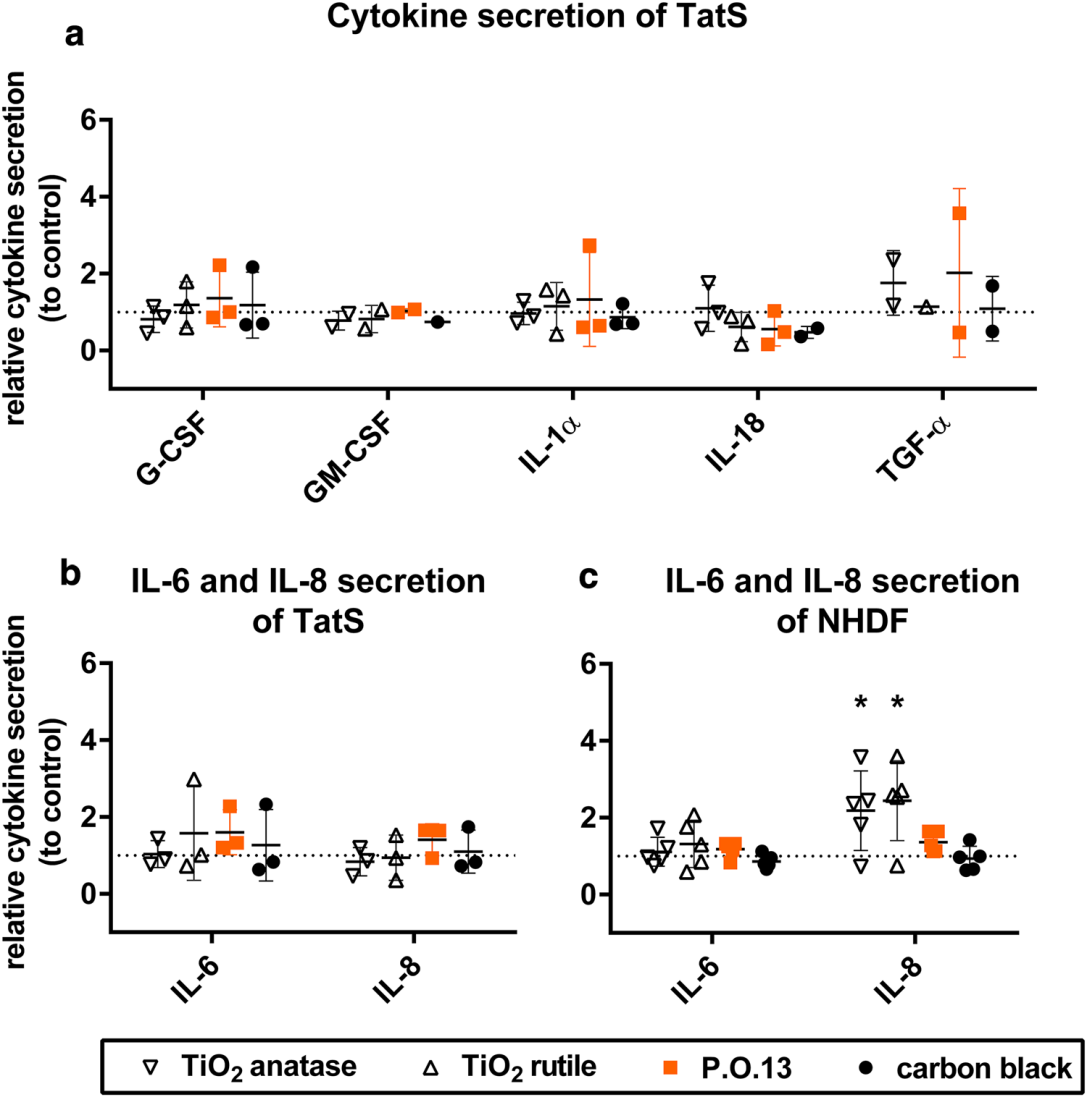
Cytokine secretion of tattooed human skin models (TatS) and normal human dermal fibroblasts (NHDF) treated with tattoo pigments. Cytokine concentrations were measured in supernatants of TatS and NHDF seeded with corresponding pigment concentrations 8 h after medium change. **a** Relative secretion of cytokines in pigmented TatS. **b, c** Relative interleukin (IL-)6 and interleukin (IL-)8 levels in supernatants of TatS and NHDF. Data were normalized by dividing each value by the value of the respective pigment-free control of the same donor. Data are displayed as mean ± SD of three TatS from different donors (*n* = 3) and NHDF from five different donors (*n* = 5). Asterisks indicate significance between substance treatments and the respective control as identified by one-way ANOVA with Dunnett’s test (* = *p* < 0.05). *G-CSF* granulocyte-colony stimulating factor, *GM-CSF* granulocyte–macrophage colony-stimulating factor, *TGF-α* transforming growth factor alpha,* TiO*_*2*_ titanium dioxide, *P.O.13* Pigment Orange 13

TatS released IL-1α, IL-6, IL-8, G-CSF and IL-18 above the limit of quantification in all replicates (Supplementary Table S4). GM-CSF and TGF-α were under or close to the quantification limit. However, the pigments had no significant effect on cytokine secretion in TatS (Fig. 5a, b).

NHDF only released detectable amounts of IL-6 and IL-8 in all replicates independent of the donor (Supplementary Table S5). IL-6 secretion of NHDF was unaffected by all pigment treatments (Fig. 5c). In contrast, TiO_2_ pigments led to a significant increase in IL-8 secretion. No effects were seen for carbon black and P.O.13 pigments.

## Discussion

Here, we established a 3D skin model for tattoo pigment research, TatS, which closely emulate tattooed skin morphology and protein expression. For the first time, an in vitro model suited to investigate the effects of the tattoo pigments carbon black, TiO_2_ and the organic azo pigment P.O.13 on human skin is reported. Moreover, the direct comparison between TatS and the NHDF monolayer cell culture revealed marked differences in cytokine secretion and cell viability upon pigment treatment.

Neither tissue viability nor cytokine secretion of TatS was altered in the presence of tattoo pigments. Due to the adjustment of pigment concentrations used with TatS that still allowed for contraction of the models during cultivation, non-toxic concentrations were already preselected. Since the concentration of P.O.13 and carbon black had to be reduced, they seem to have a greater influence on the viability of fibroblasts at higher concentrations. However, the density and weight of TiO_2_ is much higher than those of P.O.13 and especially carbon black. Hence, particle concentrations and exposed particle surfaces are much lower with TiO_2_ at the same weight. Previous studies already suggested that the surface area is the most adequate dose metric to estimate toxicity of nanoparticles (Schmid and Stoeger 2016). Here, we selected pigment concentrations according to the literature values of organic azo pigments in tattooed human skin, to be as close as possible to in vivo tattoos. These concentrations ranged from 0.6 to 9.42 mg tattoo pigment per cm^2^, with a mean value of 2.53 mg tattoo pigment per cm^2^ (Engel et al. 2008). The tattoo pigment concentration in fully-grown TatS (6 mm diameter) were around 0.6 mg/cm^2^ for P.O.13. However, pigment weight per skin surface area values are only available for organic azo pigments. Thus, the weight will be higher for TiO_2_ and lower for carbon black at the same particle concentration due to the higher or lower density compared to azo pigments, respectively. Accordingly, the pigment concentrations used for TiO_2_ and carbon black varied from the literature values and were selected by visible coloring of TatS and their viability.

The overall morphology and the abundance of development and homeostasis markers remained unchanged upon incorporation of pigments and are comparable to normal full thickness skin models that are already used in toxicology testing (Bataillon et al. 2019; Hayden et al. 2015). The presence of collagen IV as a marker of basement membrane formation and the epidermal thickness are similar to those of other human full thickness skin models made with rat tail tendon collagen cultured under the same conditions (Mieremet et al. 2019). The pigmented layer with a maximum starting depth of around 800 µm beneath the basement membrane is in range of the in vivo situation, were most pigments lay in a depth of 200–1300 µm (Ross et al. 2001).

The only published study using skin models to assess tattoo ink toxicology showed decreased viability due to in-medium treatment with full tattoo ink formulations (Bil et al. 2018). The main cytotoxic effects are attributed to the soluble ink components that reach the skin cells in this model—such as cytotoxic solvents, detergents, preservatives as well as other toxic substances (Lehner et al. 2011). The authors do not report an uptake of tattoo pigments through the insert membrane. While the uptake of in-medium applied silica nanoparticles into basal dermal cells of reconstructed human skin models has been reported elsewhere (Wills et al. 2016), this dosage is insufficient to assess the effects of tattoo pigments on human skin.

After treatment of NHDF with TiO_2_ rutile or anatase pigments in 2D, we detected an increase in the level of the proinflammatory protein IL-8. This result is in line with the increase of IL-8 expression in human epithelial colorectal adenocarcinoma (Caco-2) cells 6 h after treatment with 50 nm anatase particles in a comparable concentration (Tada-Oikawa et al. 2016). In contrast to our findings, the authors did not see an increase in IL-8 expression in Caco-2 cells after treatment with TiO_2_ particles in its rutile crystal configuration. However, the decrease in cell viability after treatment of 2D NHDF with TiO_2_ anatase in our study after 24 h matches the decrease of cell viability in tetrazolium based assays in THP-1 cells after 24 h and Caco-2 cells after 72 h when treated with anatase but also P25 and rutile TiO_2_ nanoparticles (Tada-Oikawa et al. 2016). In vivo, the secretion of the chemokine IL-8 will induce migration of granulocytes to the site of damage and stimulate phagocytosis. In tattoos, this effect is not seen after healing is completed which makes a chronic secretion of inflammatory cytokines unlikely and therefore supports a good in vivo emulation of TatS.

The discrepancy in cytotoxicity of TiO_2_ we saw between 3 and 2D cultures is well known for (nano-)particle associated cytotoxicity (Lee et al. 2009; Wills et al. 2016; Wu et al. 2017; Yu et al. 2012). In 2D cell culture, (nano-)particles are being suspended in the cell culture media. Agglomeration of particles is a common effect and results in an increased sedimentation onto the surface of cells at the bottom of the culture wells. The effective dose and hence the uptake of these nanoparticles is therefore locally increased, resulting in an overestimation of toxic effects (Cho et al. 2011). While agglomeration is visible in 3D culture, sedimentation is prevented by the extracellular matrix of 3D skin models (Wills et al. 2016). Our work therefore supports the hypothesis that the effective dose per cell is lower in the 3D culture, and 2D cell culture conditions thus lead to an overestimation of pigment toxicity at the same pigment-to-cell ratio. However, 2D experiments could still be useful to determine general toxicity by appropriate endpoints. Tattoo pigment toxicity in vivo is likely driven by size, surface properties, impurities from the manufacturing process (Regensburger et al. 2010), their interaction with UV or laser light, resulting in phototoxicity (Wamer and Yin 2010), or their degradation toward harmful substances (Hauri and Hohl 2015; Hering et al. 2018; Schreiver et al. 2015). However, research on tattoo ink associated risks requires investigating effects of single factors at a certain time. Therefore, the pigments used to establish TatS were of the highest purity available, which lowers the risk of cytotoxic effects originating from impurities. Pigment degradation is not expected under culture conditions.

The main limitation of TatS is the absence of dermal macrophages, which were shown to be a key player of tattoo persistency in rat tail skin besides fibroblasts (Baranska et al. 2018). However, the modular approach of TatS allows extending the cell types used in its construction. TatS might be combined with published human skin disease models (Bergers et al. 2016) to test the effect of tattoo pigments on diseased human skin like psoriasis (Barker et al. 2004). Also, skin cells from various areas of the body or derived from different age groups may be used (Hausmann et al. 2019). Furthermore, the small size of TatS allows testing various tattoo pigments and other ingredients at low costs.

In future, we will use TatS to investigate the interaction of pigments in the dermis with common abiotic factors such as sun light or laser irradiation. With TatS, epidermal absorption and light scattering effects of the dermal extracellular matrix will be taken into account making the resulting effects on cells in TatS more comparable to the in vivo situation—especially in terms of genotoxicity and inflammatory responses. Since only tattoo pigments are incorporated into the dermal compartment during production, TatS mimics the chronic phase of exposure when soluble ink additives are already washed out. TatS may be used as an alternative to animal experiments to evaluate pigment toxicology through appropriate endpoints as well as for basic research.

## Electronic supplementary material

Below is the link to the electronic supplementary material.

Supplementary file1 (DOCX 1073 kb)

## Abbreviations

2D: Two-dimensional
3D: Three-dimensional
DMEM: Dulbecco's modified eagle medium
DPBS: Dulbecco's phosphate-buffered saline
EDTA: Ethylenediaminetetraacetic acid
EGF: Epidermal growth factor
FBS: Fetal bovine serum
G-CSF: Granulocyte-colony stimulating factor
GM-CSF: Granulocyte–macrophage colony-stimulating factor
HEPES: 4-(2-Hydroxyethyl)-1-piperazine ethanesulfonic acid
H&E: Haematoxylin–eosin
IL: Interleukin
NHDF: Normal human dermal fibroblasts
NHK: Normal human keratinocytes
P.O.13: Pigment Orange 13
TatS: Tattooed human skin model
TiO_2_: Titanium dioxide
TGF-α: Transforming growth factor alpha
