# Supplementary material for: TatS: a novel in vitro tattooed human skin model for improved pigment toxicology research
Source: Arch Toxicol. 2020 Jul 13;94(7):2423–34. doi: 10.1007/s00204-020-02825-z (PMC7367916; doi:10.1007/s00204-020-02825-z)
Supplement: Supplementary file 1 — Supplementary file1 (DOCX 1073 kb) [file 204_2020_2825_MOESM1_ESM.docx]

**Original Article**

TatS - A Novel *in vitro* Tattooed Human Skin Model for Improved Pigment Toxicology Research

Henrik Hering^1,*^, Christian Zoschke^2^, Markus Kühn^1^, Ashish K. Gadicherla^3^, Günther Weindl^2,4^, Andreas Luch^1,2^ and Ines Schreiver^1,*^

^1^German Federal Institute for Risk Assessment (BfR), Department of Chemical and Product Safety, Berlin, Germany

^2^ Freie Universität Berlin, Institute of Pharmacy (Pharmacology & Toxicology), Berlin, Germany

^3^German Federal Institute for Risk Assessment (BfR), Department of Biological Safety, Berlin, Germany

^4^University of Bonn, Section of Pharmacology and Toxicology, Pharmaceutical Institute, Bonn, Germany

^*^Corresponding authors: [Henrik.Hering@bfr.bund.de](mailto:Henrik.Hering@bfr.bund.de), Ines.Schreiver@bfr.bund.de

Keywords: rutile, anatase, carbon black, Pigment Orange 13, tissue engineering, tattooing

**Supplementary Tables**

**Supplementary Table S1: Relative fluorescence intensities of complete TatS in the PrestoBlue viability assay**

|  | **Relative fluorescence intensity (AU)** | | |  | |  |
| --- | --- | --- | --- | --- | --- | --- |
| Treatment^a^ | I | II | III | Mean | SD |  |
| control^b^ | 61,520 | 64,661 | 61,662 | 62,614 | 1,774 |  |
| TiO_2_ anatase | 63,362 | 44,857 | 58,441 | 55,553 | 9,585 |  |
| TiO_2_ rutile | 59,440 | 65,904 | 52,735 | 59,360 | 6,585 |  |
| P.O.13 | 60,250 | 69,978 | 35,711 | 55,313 | 17,659 |  |
| carbon black | 56,853 | 88,095 | 62,680 | 69,209 | 16,613 |  |

^a^ I-III show biological replicates (cells from different donors)

^b^ Control: TatS without pigment treatment

Abbreviations: TatS = tattooed human skin model, AU = arbitrary unit, P.O.13 = Pigment Orange 13; TiO_2_ = titanium dioxide, NHDF = normal human dermal fibroblasts

**Supplementary Table S2: Relative fluorescence intensities of dermal TatS layers in the PrestoBlue viability assay**

|  | **Relative fluorescence intensity (AU)** | | |  | |  |
| --- | --- | --- | --- | --- | --- | --- |
| Treatment^a^ | I | II | III | Mean | SD |  |
| control^b^ | 63,578 | 34,772 | 55,958 | 51,436 | 14,926 |  |
| TiO_2_ anatase | 63,171 | 51,544 | 44,197 | 52,971 | 9,567 |  |
| TiO_2_ rutile | 62,266 | 57,625 | 53,233 | 57,708 | 4,517 |  |
| P.O.13 | 63,039 | 38,182 | 41,395 | 47,539 | 13,520 |  |
| carbon black | 62,843 | 34,170 | 45,635 | 47,549 | 14,432 |  |

^a^ I-III show biological replicates (cells from different donors)

^b^ Control: NHDF without pigment treatment

Abbreviations: TatS = tattooed human skin model, AU = arbitrary unit, P.O.13 = Pigment Orange 13; TiO_2_ = titanium dioxide

**Supplementary Table S3: Relative fluorescence intensities of NHDF in the PrestoBlue viability assay**

|  | **Relative fluorescence intensity (AU)** | | | | |  |  |
| --- | --- | --- | --- | --- | --- | --- | --- |
| Treatment^a^ | I | II | III | IV | V | Mean | SD |
| control^b^ | 47,013 | 59,734 | 46,896 | 58,217 | 46,609 | 51,693 | 6,670 |
| TiO_2_ anatase | 24,434 | 40,917 | 18,197 | 53,455 | 41,879 | 35,776 | 14,265 |
| TiO_2_ rutile | 42,421 | 54,077 | 32,899 | 55,407 | 45,448 | 46,050 | 9,199 |
| P.O.13 | 46,813 | 58,562 | 44,039 | 49,629 | 46,114 | 49,031 | 5,691 |
| carbon black | 43,563 | 58,634 | 42,226 | 57,642 | 45,006 | 49,414 | 8,032 |

^a^ I-V show biological replicates (NHDF from different donors)

^b^ Control: NHDF without pigment treatment

Abbreviations: NHDF = Normal human dermal fibroblasts, AU = arbitrary unit, P.O.13 = Pigment Orange 13; TiO_2_ = titanium dioxide

**Supplementary Table S4: Cytokine secretion of TatS at day 21**

|  | **Cytokine concentrations in the supernatant (pg/ml)** | | | | | | |
| --- | --- | --- | --- | --- | --- | --- | --- |
| cytokine | G-CSF | GM-CSF | IL-1α | IL-6 | IL-8 | IL-18 | TGF-α |
| LOQ | <1.24 | <0.81 | <1.59 | <1.65 | <0.72 | <1.69 | <1.49 |
| control | 93.4 | < LOQ | 5.9 | 1,018.7 | 845.1 | 52.0 | < LOQ |
|  | 1663.5 | 4.4 | 22.6 | 895.4 | 962.9 | 23.9 | 19.5 |
|  | 158.1 | 3.5 | 8.1 | 734.6 | 1,511.3 | 3.6 | 7.6 |
| TiO_2_ anatase | 105.1 | < LOQ | 5.3 | 1,458.9 | 1,002.0 | 90.8 | < LOQ |
|  | 741.9 | 2.7 | 16.1 | 686.7 | 441.9 | 13.4 | 45.8 |
|  | 136.5 | 3.4 | 10.3 | 661.6 | 1,288.1 | 3.5 | 8.9 |
| TiO_2_ rutile | 167.9 | < LOQ | 9.3 | 3,038.7 | 1,292.3 | 46.4 | < LOQ |
|  | 996.1 | 2.5 | 9.8 | 654.1 | 343.3 | 4.2 | < LOQ |
|  | 182.7 | 3.8 | 11.5 | 746.6 | 1,408.3 | 2.7 | 8.7 |
| P.O.13 | 80.5 | < LOQ | 3.8 | 1,223.8 | 787.2 | 8.5 | < LOQ |
|  | 3,690.1 | 4.4 | 13.7 | 2,038.0 | 1,588.3 | 11.5 | 69.7 |
|  | 157.5 | 3.8 | 22.1 | 974.7 | 2,487.6 | 3.7 | 3.6 |
| carbon black | 202.7 | < LOQ | 4.2 | 2,375.1 | 1,474.6 | 30.0 | < LOQ |
|  | 1,152.2 | 3.3 | 27.4 | 744.6 | 794.0 | 8.7 | 32.9 |
|  | 106.2 | < LOQ | 5.5 | 462.2 | 1,094.6 | < LOQ | 3.8 |

Abbreviations: TatS = tattooed human skin model, LOQ = Limit of quantification, G-CSF = granulocyte-colony stimulating factor, GM-CSF = granulocyte-macrophage colony-stimulating factor, IL = interleukin, TGF-α = transforming growth factor alpha, TiO_2_ = titanium dioxide, P.O.13 = Pigment Orange 13

**Supplementary Table S5: Cytokine secretion of NHDF**

|  | **Cytokine concentrations in the supernatant (pg/ml)** | | | | | | |
| --- | --- | --- | --- | --- | --- | --- | --- |
| cytokine | G-CSF | GM-CSF | IL-1α | IL-6 | IL-8 | IL-18 | TGF-α |
| LOQ | <1.24 | <0.81 | <1.59 | <1.65 | <0.72 | <1.69 | <1.49 |
| control | 4.5 | < LOQ | 18.0 | 1,884.8 | 277.7 | 3.8 | 4.7 |
|  | 7.9 | < LOQ | 42.5 | 1,418.5 | 204.0 | 2.9 | 4.7 |
|  | < LOQ | < LOQ | < LOQ | 123.6 | 74.5 | < LOQ | < LOQ |
|  | < LOQ | 0.9 | 3.5 | 1,219.7 | 581.1 | < LOQ | 4.4 |
|  | 6.0 | 7.3 | 22.1 | 1,742.3 | 1,229.8 | 6.7 | 13.3 |
| TiO_2_ anatase | 4.7 | < LOQ | 12.5 | 1,731.7 | 678.6 | < LOQ | < LOQ |
|  | 5.5 | < LOQ | 8.7 | 1,366.4 | 481.3 | 3.1 | 4.3 |
|  | 3.1 | < LOQ | 3.3 | 150.1 | 266.3 | < LOQ | < LOQ |
|  | < LOQ | < LOQ | < LOQ | 865.2 | 347.2 | < LOQ | < LOQ |
|  | 7.0 | 0.9 | 1.7 | 2,487.3 | 1,827.3 | 2.3 | 4.7 |
| TiO_2_ rutile | 4.3 | < LOQ | 9.4 | 1,116.0 | 209.5 | < LOQ | < LOQ |
|  | 4.3 | < LOQ | 11.6 | 1,208.3 | 517.8 | 5.4 | 6.2 |
|  | 3.1 | < LOQ | 7.6 | 161.3 | 268.7 | 3.2 | 4.7 |
|  | < LOQ | 0.9 | 2.3 | 1,276.7 | 641.7 | 2.9 | 5.3 |
|  | 4.6 | 1.0 | < LOQ | 2,685.7 | 2,662.5 | 1.8 | 7.6 |
| P.O.13 | 7.5 | < LOQ | 21.0 | 2,361.4 | 309.7 | 4.4 | < LOQ |
|  | 4.7 | 12.9 | 11.7 | 1,171.4 | 261.6 | 6.0 | < LOQ |
|  | 2.3 | < LOQ | 3.3 | 163.7 | 122.3 | 2.8 | < LOQ |
|  | < LOQ | < LOQ | < LOQ | 1,061.2 | 419.2 | < LOQ | 5.9 |
|  | < LOQ | 1.0 | < LOQ | 1,751.7 | 1,355.1 | < LOQ | 4.0 |
| carbon black | 4.3 | < LOQ | 18.7 | 1,243.7 | 175.3 | 2.9 | 6.2 |
|  | < LOQ | < LOQ | 5.2 | 1,138.8 | 203.0 | 3.0 | < LOQ |
|  | < LOQ | < LOQ | < LOQ | 138.7 | 105.9 | < LOQ | 4.0 |
|  | < LOQ | < LOQ | < LOQ | 867.7 | 312.6 | < LOQ | 3.3 |
|  | < LOQ | < LOQ | < LOQ | 1,606.3 | 861.6 | 3.4 | 2.9 |

Abbreviations: NHDF = normal human dermal fibroblasts, LOQ = Limit of quantification; G-CSF = granulocyte-colony stimulating factor, GM-CSF = granulocyte-macrophage colony-stimulating factor, IL = interleukin, TGF-α = transforming growth factor alpha, TiO_2_ = titanium dioxide, P.O.13 = Pigment Orange 13

**Supplementary Figures**


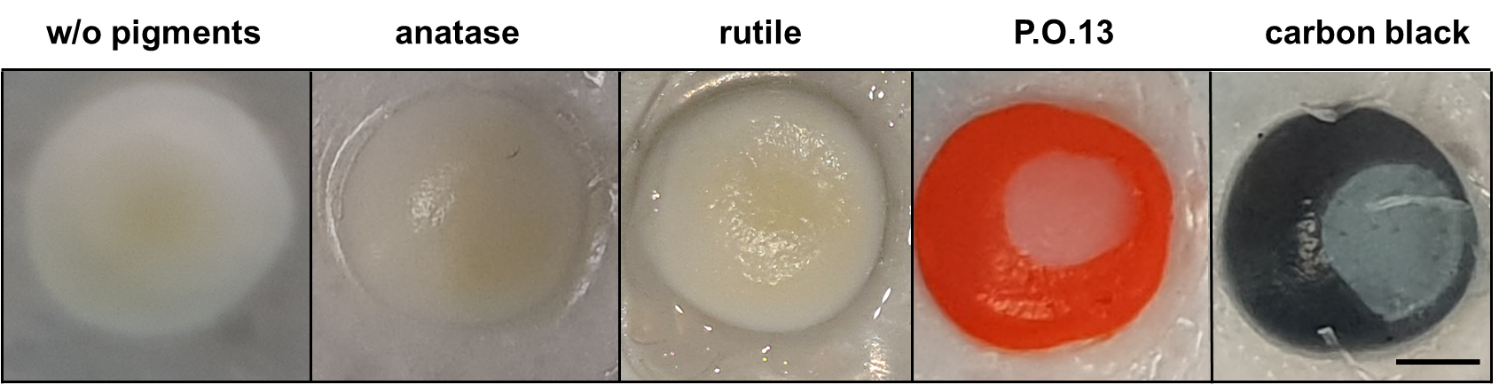


**Supplementary Fig. S1** Macroscopic pictures of tattooed human skin models (TatS). Pictures of TatS were taken at the end of culture (day 21). Pigmented TatS show good coloring, comparable to a tattoo *in vivo*, especially with P.O.13 and carbon black. Pigment-free dermal layers are visible in the center of the models. Black bar equals 2 mm. The diameter of TatS is around 6 mm. Abbreviations: w/o = without, TiO_2_ = titanium dioxide, P.O.13 = Pigment Orange 13


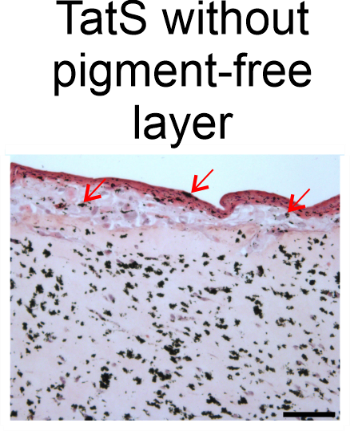


**Supplementary Fig. S2** TatS without pigment-free layer showed ectopic pigments in the epidermal layer. Haematoxylin and eosin staining of TatS sections revealed pigment migration from the dermal collagen layer into the epidermis during development of TatS (red arrow). The example shows a preliminary carbon black model. Black bar equals 100 µm
